# “If you are here at the clinic, you do not know how many people need help in the community”: Perspectives of home-based HIV services from health care workers in rural KwaZulu-Natal, South Africa in the era of universal test-and-treat

**DOI:** 10.1371/journal.pone.0202473

**Published:** 2018-11-09

**Authors:** Delphine Perriat, Mélanie Plazy, Dumile Gumede, Sylvie Boyer, Deenan Pillay, François Dabis, Janet Seeley, Joanna Orne-Gliemann, for the ANRS 12249 TasP Study Group

**Affiliations:** 1 Univ. Bordeaux, Inserm, Bordeaux Population Health Research Center, Bordeaux, France; 2 Inserm, ISPED, Bordeaux Population Health Research Center, Bordeaux, France; 3 Africa Health Research Institute, KwaZulu-Natal, South Africa; 4 Aix Marseille Univ, INSERM, IRD, SESSTIM, Sciences Economiques & Sociales de la Santé & Traitement de l’Information Médicale, Marseille, France; 5 University College London, Division of Infection and Immunity, London, United Kingdom; 6 London School of Hygiene and Tropical Medicine, London, United Kingdom; Johns Hopkins University, UNITED STATES

## Abstract

**Background:**

Limited engagement in clinic-based care is affecting the HIV response. We explored the field experiences and perceptions of local health care workers regarding home-based strategies as opportunities to improve the cascade of care of people living with HIV in rural South Africa as part of a Universal Test-and-Treat approach.

**Methods:**

In Hlabisa sub-district, home-based HIV services, including rapid HIV testing and counselling, and support for linkage to and retention in clinic-based HIV care, were implemented by health care workers within the ANRS 12249 Treatment-as-Prevention (TasP) trial. From April to July 2016, we conducted a mixed-methods study among health care workers from the TasP trial and from local government clinics, using self-administrated questionnaires (n = 90 in the TasP trial, n = 56 in government clinics), semi-structured interviews (n = 13 in the TasP trial, n = 5 in government clinics) and three focus group discussions (n = 6–10 health care workers of the TasP trial per group). Descriptive statistics were used for quantitative data and qualitative data were analysed thematically.

**Results:**

More than 90% of health care workers assessed home-based testing and support for linkage to care as feasible and acceptable by the population they serve. Many health care workers underlined how home visits could facilitate reaching people who had slipped through the cracks of the clinic-based health care system and encourage them to successfully access care. Health care workers however expressed concerns about the ability of home-based services to answer the HIV care needs of all community members, including people working outside their home during the day or those who fear HIV-related stigmatization. Overall, health care workers encouraged policy-makers to more formally integrate home-based services in the local health system. They promoted reshaping the disease-specific and care-oriented services towards more comprehensive goals.

**Conclusion:**

Because home-based services allow identification of people early during their infection and encourage them to take actions leading to viral suppression, HCWs assessed them as valuable components within the panel of UTT interventions, aiming to reach the 90-90-90 UNAIDS targets, especially in the rural Southern African region.

**Trial registration:**

The registration number of the ANRS 12249 TasP trial on ClinicalTrials.gov is NCT01509508.

## Introduction

Coverage of HIV testing and treatment remains sub-optimal, especially in low–income countries, which carry the heaviest HIV burden worldwide [1]. To fast track the response to the pandemic, UNAIDS in 2014 set out the 90-90-90 targets (namely, that 90% of people living with HIV know their status; that 90% of all people with diagnosed HIV infection receive sustained antiretroviral treatment (ART); and that 90% of all people receiving ART have viral suppression). In 2015, the World Health Organization (WHO) recommended ART for all people living with HIV [2]. In this context, the Universal Test and Treat (UTT) strategy aims at offering regular HIV testing and counselling to an entire population, and ART to all those living with HIV.

Several research projects, including randomized controlled trials in Southern and Eastern Africa, are currently accumulating evidence on the feasibility, acceptability and effectiveness of UTT strategies to fight the HIV epidemic [3]. UTT can include a large and diverse range of HIV-related services at each step of the HIV care cascade, starting from community mobilization and prevention, to testing and linkage to care, through to treatment and retention in care. These services can be delivered at home, within the community and/or in clinics [4].

Historically and worldwide, the HIV care model has been mainly facility-based, with patients presenting themselves for HIV testing and treatment [5]. Yet, access to facility-based HIV care has been sub-optimal especially in rural and resource-constrained settings [6], and the role of home-based services has been increasingly promoted [7]. Home-based services including the provision of HIV testing and counselling within households, or home visits to support linkage to care and retention have been shown to be highly acceptable by patients, families, communities and health care workers (HCWs) [8] and to improve the uptake of HIV testing [9, 10], linkage to care and treatment adherence rates [11] while being three to four times cheaper than clinic-based approaches [12, 13]. However, the high initial operating costs, especially in terms of human resources and logistics have deterred decision-makers from officially integrating home-based services in the local and national health care systems. Home-based services have thus been facing various challenges such as inadequate training, support and supervision of HCWs [14], poor service coverage, due to the uneven geographical distribution of HCWs [15], and poor recognition of the value of HCW’s work [7, 16]. Regardless, WHO recently reemphasized the prospects of home-based models for HIV control, especially in rural and highly HIV prevalent areas involving lay care providers [17–20].

South Africa is facing the biggest HIV epidemic in the world (7 million of people are living with HIV) [21]. Despite intensive efforts, HIV testing and specifically repeat testing, linkage and retention in care in the local and national HIV care programs remain sub-optimal [11, 22]. The national government has been supporting local initiatives for a diversification of health interventions including home-based HIV services, especially in rural areas [13, 23–25]. However home-based services have not yet been standardized and integrated within the national health system [26]. In September 2016, South Africa adopted a policy of providing ART to everyone living with HIV, posing a challenge to the current health system. Home-based services could be a valuable component to address the increased demands for HIV care provision [27, 28].

The long-term benefits and sustainability of home-based services as part of a UTT strategy are being explored in population-based trials currently ongoing in Southern and Eastern Africa [3]. Among them, the first to yield results was the ANRS 12249 TasP (Treatment-as-Prevention) trial, a cluster-randomized trial implemented in rural KwaZulu-Natal, South Africa [29, 30]. The UTT strategy implemented within the TasP trial included a home-based approach for HIV testing and support for linkage and retention in care. It was highly successful in terms of HIV testing coverage within these poor and disadvantaged rural communities (92% home-based HIV testing and counselling uptake among those offered the services) [31] and also contributed to significantly increase rates of linkage to care [32]. While these findings suggest support for the large-scale implementation of home-based services, implementation of this approach demands the buy-in of frontline HCWs who will oversee delivery of those services. The TasP trial provides a unique opportunity to investigate how HCWs view home-based services in the context of a UTT strategy. We explored the experiences and perceptions of local HCWs regarding home-based HIV services as strategic components of the arsenal of UTT interventions, aiming at improving the cascade of care of people living with HIV in rural South Africa.

## Methods

### Study setting/context: The ANRS 12249 TasP trial

The TasP trial was implemented by the Africa Health Research Institute (AHRI) (former Africa Centre for Population Health) from March 2012 to June 2016 in Hlabisa sub-district (northern KwaZulu-Natal), in a rural area with approximately 28,000 isiZulu-speaking resident adults [33]. The HIV prevalence in the sub-district is one of the highest in the world, with around 29% of adults infected with HIV [34]. The trial aimed to investigate whether immediate ART initiation offered to all HIV-positive individuals, identified through home-based HIV testing, reduced HIV incidence in the area.

**Fig 1:**
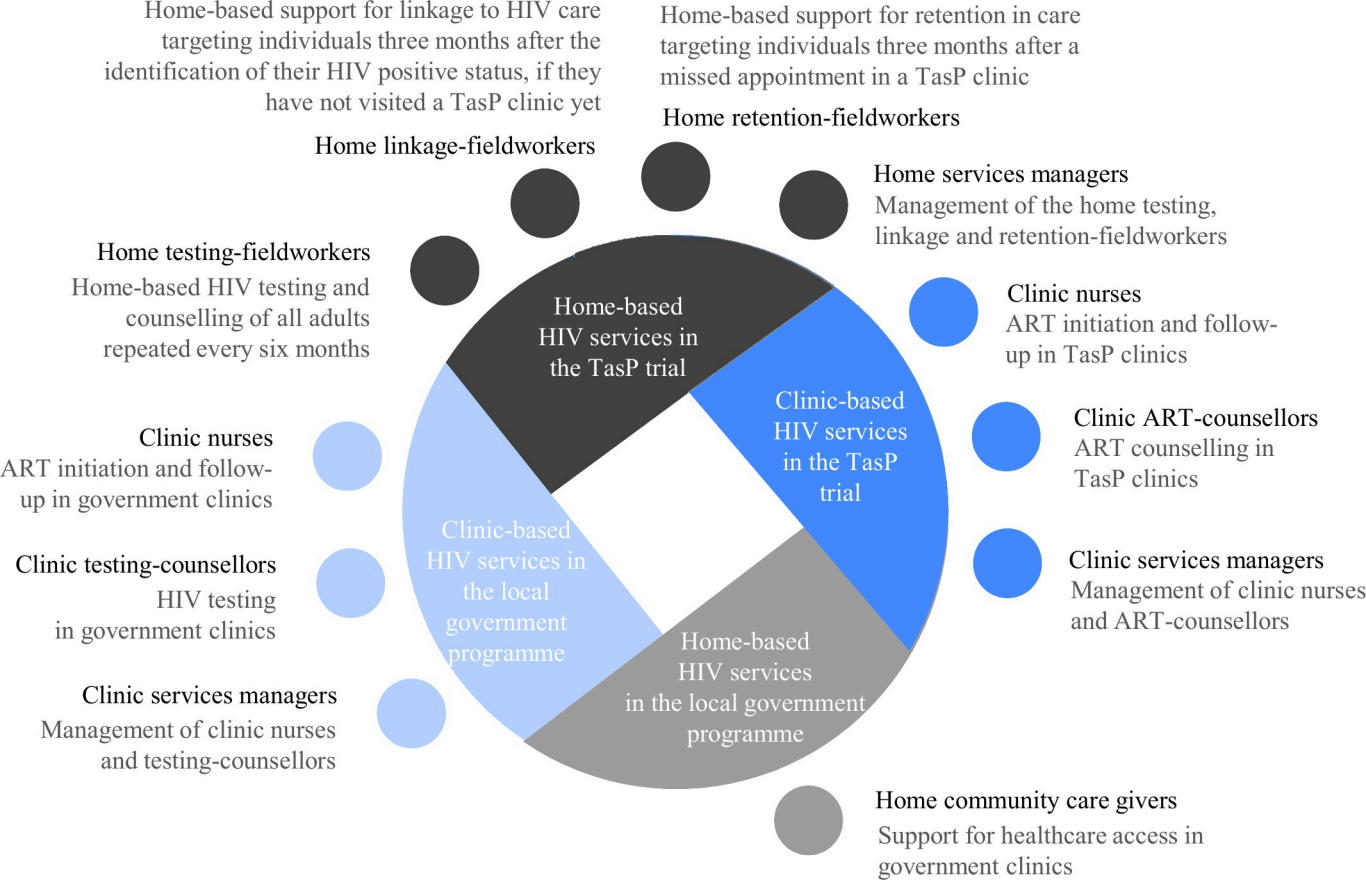
Study population. ART: antiretroviral treatment, TasP: ANRS 12249 Treatment-as-Prevention trial.

The UTT strategy implemented in the TasP trial had two main components: 1. Home-based HIV testing and counselling offered every six months to all adults of 16 years and above who reside in the trial area, and referral of those identified HIV+ to local trial clinics for HIV care, 2. ART initiation in local trial clinics (initiation regardless of CD4 count in the intervention arm, vs initiation according to the South African guidelines, i.e. at a CD4 count ≤ 350 cells/μL prior January 2015, then CD4 count ≤ 500 cells/μL in the control arm). Additional services were implemented for HIV-positive individuals, namely: phone and home-based support for linkage to care targeting individuals who had not visited a trial clinic within three months after identification of their HIV positive status; and phone and home-based support for retention in care targeting individuals three months after a missed appointment in a trial clinic. These services were delivered by a wide range of skilled HCWs (Fig 1).

The trial area was also served by the HIV treatment and care programme in the local government clinics of Hlabisa sub-district, in which counsellors offer HIV testing and nurses offer treatment to people in local government clinics [35]. Additionally, a network of community volunteers with limited training in healthcare, referred to as Community Care Givers (CCGs), regularly visit people’s homes to encourage them to visit the clinics to get healthcare, including HIV-related healthcare [36, 37].

### Study design, study tools, study population and sample

We conducted a cross-sectional mixed methods study in the TasP trial area in South Africa. The quantitative component of the study, which was conducted in April 2016 aimed at measuring the global appreciation of HCWs regarding the acceptability and feasibility of home-based HIV services as part of a UTT strategy. The qualitative component conducted between May and July 2016 aimed at describing the reasons behind such perceptions as well and the specific personal experiences of HCWs [38]. All HCWs involved in the TasP trial service delivery (n = 104) and working in the HIV services of the 17 government primary health care clinics in Hlabisa sub district (n = 78) were invited to participate in the quantitative study. The qualitative component of the study consisted of semi-structured individual interviews (SSI) and focus-group discussions (FGD). Participants to the qualitative component were selected among the staff met during the quantitative component of the study, and based on three main criteria (age, years of experience, high versus low clinic volume) to capture diverse professional contexts. HCWs belonged to three different groups depending on the services they delivered: home-based HIV services in the TasP trial, clinic-based HIV services in the TasP trial or clinic-based HIV services in the local government programme (Fig 1). A total of 18 SSIs (13 with TasP staff and 5 with government staff) and three FGDs with TasP staff (6 to 10 participants) were conducted (Table 1).

**Table 1:** Personal information in quantitative research participants (N = 146).

	N (%)
**Gender** (N = 146)	
Male	65 (24%)
Female	81 (76%)
**Median age [IQR] (years)** (N = 134)	34 [30–40]
**Professional role** (N = 146)	
TasP home testing-fieldworkers	38
TasP home linkage-fieldworkers	6
TasP home retention-fieldworkers	8
TasP home services managers	5
TasP clinic nurses	15
TasP clinic ART-counsellors	15
TasP clinic services managers	3
Government clinic nurses	40
Government clinic testing-counsellors	16

IQR: interquartile range

### Data collection

Quantitative component: Self-administrated structured paper-based questionnaires were distributed by the researchers to the staff working in the HIV services of the government clinics and to the staff of the TasP trial during their respective staff meetings in their workplace. Logistical help was provided by AHRI to inform the Department of Health (DoH) and TasP managers of the study, arrange for the staff availability to participate in the study and introduce the researchers to the staff. Prior to questionnaire completion, the researchers briefly presented the study to participants, highlighting that completing a questionnaire was voluntary and anonymous. The completion of a questionnaire lasted for up to 30 minutes. Participants then inserted the questionnaire in a closed box to preserve anonymity. All questionnaires were in English and had the same structure: (1) a section on demographic content (2) questions on the experience of HCWs in providing HIV services (the content of this section was adapted to the specificities of the staff profession), (3) questions eliciting their perceptions of various UTT interventions, and (4) questions measuring their job satisfaction. The instrument was piloted with staff of the TasP trial, which resulted in some minor changes related to item wording. Data were captured using the Research Electronic Data Capture (REDCap) software.

Qualitative component: Four local experienced qualitative interviewers were recruited to carry out FGDs and SSIs in isiZulu. They received specific training on interviewing techniques with staff members, to minimize bias from respondents when questioned about the institution they work for. SSIs lasted for 60 to 90 minutes, and FGDs for 120 to 160 minutes. They were held either in a closed meeting room at AHRI for the TasP staff, or at a place of convenience for the participants from the government clinics. Semi-structured guides for the SSIs and FGDs were designed to fit the specificities of the range of professions interviewed. Participants were asked about their roles and motivations in their work, the challenges they faced and their perceptions of the strengths and weaknesses of the interventions delivered in the TasP trial, as well as their opinion on the scale-up of those interventions to the whole of Hlabisa sub-district and beyond. Data were audio recorded, transcribed in isiZulu and translated to English. The quality of the translations was checked by the most experienced qualitative interviewer. During the whole data collection, interviewers were supervised and coached by researchers who provided them with professional support to manage their tasks and facilitate fieldwork.

### Analysis process

Quantitative component: This publication explores quantitative data extracted from the third section of the study questionnaire, regarding the perceptions of HCWs on home-based HIV services, including HIV testing and personalized support for linkage to care. Respondents rated the degree to which they agreed or disagreed with statements using a five point ordinal Likert scale (totally disagree, partially disagree, no opinion, partially agree, totally agree). Results were analysed using R software version 3.0.1.

Qualitative component: An inductive approach based on descriptive thematic coding was used to explore the qualitative data. All transcripts were entered in a qualitative data analysis management software (NVivo version 11, QSR International Pty Ltd., Doncaster, Victoria, 3108, Australia). Codebooks were developed using an iterative process: initial codes matched the broad research themes that we used in the interview and focus group discussion guides, then were expanded with codes, drawn from the data, reflecting the participants’ own terms and semantics, and discussed within the team, including qualitative interviewers and researchers, to triangulate input. The hierarchical coding framework contained five main codes, with four levels of branching each, and a total of over 60 sub-codes. Examples of main codes are: organization of resources to meet the HIV health needs of the community, ability of the organization to deliver HIV services, attitudes and perceptions of community members regarding seeking and accessing care. This publication focuses on the data coded using these sub-codes on three types of home-based services: HIV testing and counselling, support for linkage to care, and support for retention in care.

### Ethics

Formal ethics approval was provided by the Biomedical Research Ethical Council (BREC) of the University of KwaZulu-Natal on March 18, 2016. All participants voluntarily gave their informed written consent.

## Results

Overall, 146 quantitative questionnaires were completed (overall response rate 80%: 86% for TasP staff and 72% for government staff). Reasons for non-completion of the questionnaires were mainly annual or sick leave and unavailability at the time of the clinic visit. Only two were explicit refusals. As the South African health workforce is predominantly female (over 90% in KwaZulu-Natal [39]), most respondents were women in both the quantitative (n = 81, 76%) and the qualitative (n = 38, 92%) studies. Participants ranged in age from 25 to 74 years old in the quantitative study (median age = 34 years, interquartile range (IQR) = [30–40]) (Table 1) and from 25 to 72 years old in the qualitative study (median age = 37 years, IQR = [32–53]) (Table 2 and Table 3).

**Table 2:** Personal information in research participants of qualitative semi-structured interviews (N = 18).

Position/Cadre	Sex	Age (years)	Experience in HIV care (years)
**Home-based HIV services in the TasP trial**			
Home linkage-fieldworker 1	F	40	9
Home linkage-fieldworker 2	F	53	10
Home retention-fieldworker	F	41	14
Home services manager 1	F	32	3
Home services manager 2	F	42	12
**Clinic-based HIV services in the TasP trial**			
Clinic nurse 1	F	35	8
Clinic nurse 2	F	53	8
Clinic nurse 3	F	34	6
Clinic nurse 4	F	71	10
Clinic ART counsellor 1	F	46	16
Clinic ART counsellor 2	F	31	9
Clinic trial manager 1	F	39	8
Clinic trial manager 2	F	44	11
**Clinic-based HIV services in the local government programme**			
Clinic nurse 1	F	47	17
Clinic nurse 2	F	63	7
Clinic nurse 3	F	50	6
Clinic services manager 1	F	31	3
Clinic services manager 2	F	56	17

F: female, M: male

**Table 3:** Personal information in research participants of qualitative focus group discussions (N = 23).

Position/Cadre	Number of participants	Sex	Median age [range] (years)	Median experience in HIV care [range] (years)
**Home-based HIV services in the TasP trial**				
Home testing-fieldworkers	10	9F, 1M	31 [26–41]	6 [3–13]
**Clinic-based HIV services in the TasP trial**				
Clinic nurses	7	6F, 1M	46 [34–71]	7 [5–9]
Clinic ART counsellors	6	5F, 1M	45 [29–58]	11 [6–14]

F: female, M: male

### Home-based services have the potential to draw into care people who have slipped through the cracks of the existing health care system

#### Home-based services are perceived as highly acceptable and very convenient

**Fig 2:**
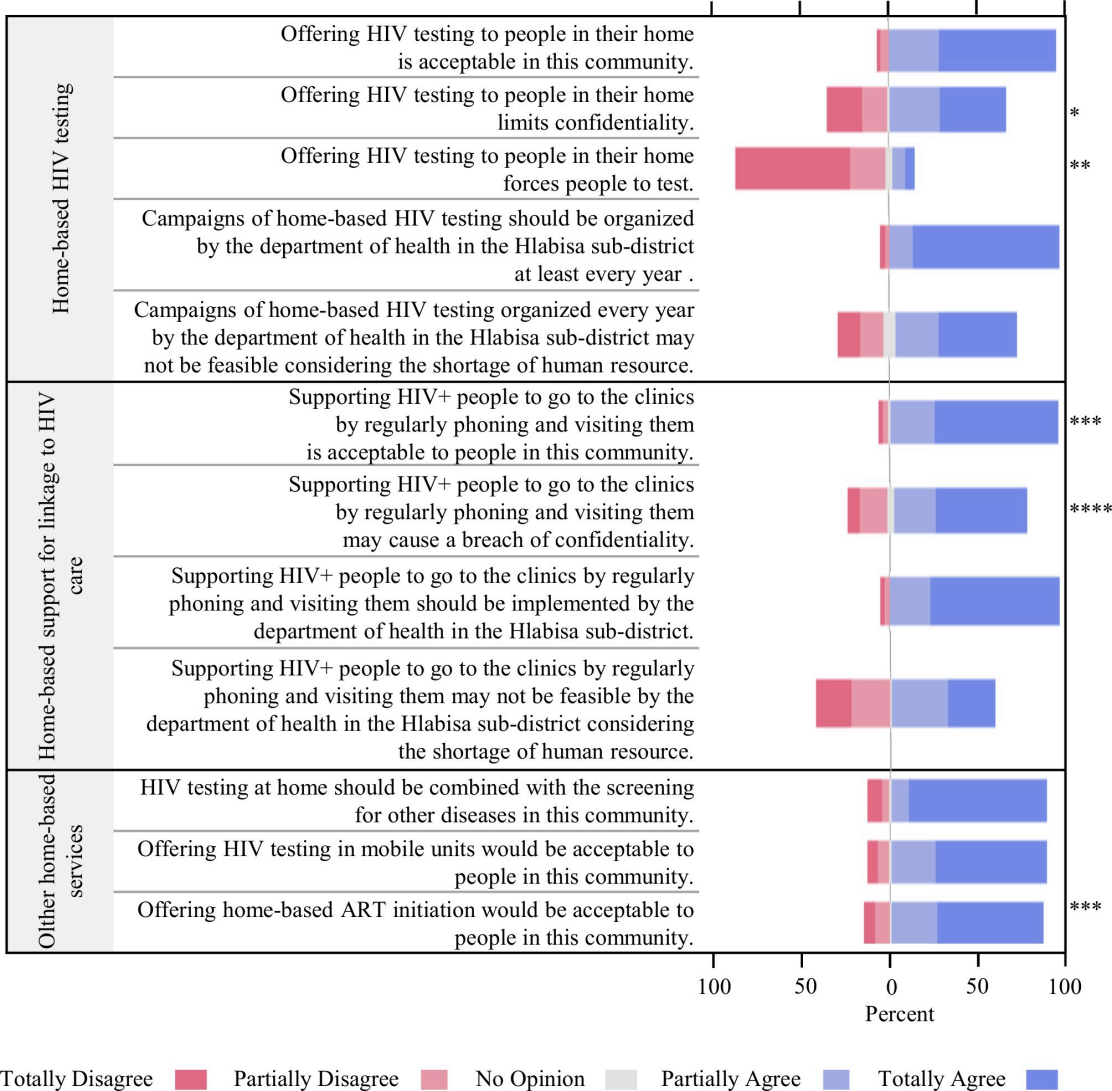
Perceptions of HCWs on HIV services delivery (n = 146). ART: antiretroviral treatment, * The variable has 2 missing values, ** The variable has 1 missing value, *** The variable has 3 missing values, **** The variable has 4 missing values.

Over 90% of the quantitative study participants affirmed that home-based services are acceptable by the population they serve (Fig 2). HCWs who delivered such services within the TasP trial explained during the qualitative interviews that community members were usually agreeable to them entering their household and appreciated receiving HIV services in their homes.

*“People were happy that we visited them in their homes; giving them information, testing them for HIV*. *They were also happy to access the trial clinics, which were close by” (SSI, TasP clinic ART-counsellor 2)*

*“I can say that people from rural areas are not rude*. *[…] It never happened that we would arrive at a participant’s home, and the participant would refuse to talk with us. People from rural areas have respect.” (SSI, TasP home linkage-fieldworker 2)*

Additionally, home-based services were seen as a possible means to overcome some common structural and individual barriers to facility-based health services: people are spared walking long distances, paying for transport to visit a clinic, or queuing for hours in the waiting room and risking to be seen by community members.

*“According to me*, *it* (meaning home-based HIV testing) *is a good thing*, *because some (trial) participants were afraid to go to the [government] clinic to get tested*, *and to queue for a long time to be seen*. *It happens that people are sick and afraid to go to clinic to know his/her status*. *But when we go to people’s home (in the TasP trial)*, *we request everyone individually to come and test with us*. *We don’t choose*. *Then the participants will know their status*.*” (FGD*, *TasP home testing-fieldworkers*, *participant 6)*

#### Home-based services enable a strong support for HIV care due to family closeness and connectedness with HCWs

All TasP HCWs who delivered services at home mentioned during the interviews the role of the family in people’s acceptance of HIV services. They observed powerful group dynamics at home, with family members encouraging each other.

*“What’s good about the TasP trial is that, when we arrive at participants’ home, we sometimes find people that don’t want to be tested for HIV*. *But if they see us testing one of their family members, they change their minds and push each other to be tested […]. It becomes easier for them” (FGD, TasP home testing-fieldworkers, participant 7)*

Many HCWs emphasized the strong interpersonal skills they had to display to engage in extended one-to-one talks with people in their homes. They expressed the rewarding feeling they got from helping people most in need.

*“That’s why I love linkage activities. Because it has helped many people who were sitting at home*. *[…] Once, a participant (to the TasP trial) was happy and said to me “Wow, you are the one who helped me. I was dead and afraid, but now I am alive and not afraid.” (SSI, TasP home linkage-fieldworker 2)*

*“You find that a grandmother’s child has been helped because you encouraged him/her to go to the clinic*. *The grandmother would have failed on various occasions to convince her child to go to the clinic […]. But then you come in their home and help the child. Then the grandparents will love you because they see the results of your work.” (SSI, TasP home linkage-fieldworker 2)*

### Home-based services are conducive to promoting entry and retention in HIV care

Home-based HIV services were perceived as promising add-ons to boost the HIV care cascade. During the qualitative interviews, several HCWs explained that HIV testing at home allowed people to learn about their positive HIV status, even those that wouldn’t have voluntarily sought HIV testing. Others underlined the potential of home visits to diagnose people early in the course of their infection, therefore empowering them to seek healthcare. Individuals receive personalized support and are encouraged to build enough self-confidence to enter and be retained in care.

*“I loved home testing because some people are not aware that they are HIV-positive*. *By testing for HIV at home, they get to know their statuses earlier. This allows them to make informed decisions on how they will continue living their lives.” (SSI, TasP clinic nurse 4)*

*“Some trial participants were referred by home testing-fieldworkers for counselling during home visits. They would realize that, the reason why they are sick is that they now have the virus. They find out about it when they are told there [at home]*. *Then they come for help [at the clinic]. You can see that, as time goes by, people who have not yet accepted [their positive HIV status] start saying: “Oh! All these illnesses that I had, it was because I was infected by HIV and not aware of it. I am now okay and feel well.” (FGD, TasP clinic ART-counsellors, participant 1)*

Additionally, many TasP HCWs noted that providing respectful HIV services at home contributed to building people’s trust towards clinic-based health care providers.

*“People* (meaning some trial participants) *say that they come to the trial clinics because of the way home testing-fieldworkers showed them care during the home visits*. *They assumed that they would receive the same care at the clinic than the one they received from the fieldworkers at home*. *The way fieldworkers behave and care for them is one of the reasons why they come to the clinics*.*” (FGD*, *TasP clinic ART counsellors*, *participant 2)*

Government HCWs specifically mentioned how teaming up with CCGs, who move about in the community, including to people’s homes, contributes to tracking the lost-to-care populations to encourage them to use clinic-based services.

### Home-based services are not a magic bullet

#### Home-based services don’t reach everyone

Overall, TasP home fieldworkers explained that the main reason for not being able to offer home services to community members was that people were not present in their households at the time of the home visits (e.g. people were working, especially men, were at school or had moved away to another area). Home fieldworkers underlined that home visits were never refused when prior agreement had been provided during a phone call.

*“We had a challenge of finding males because they were not found in their homes during the day. […]. But those who were at home at the time of home visit, we were able to find them. There isn’t a difference in testing [between males and females]*. *Once you find the participant, s/he will accept [as] they are taught that they should take care of themselves.”(SSI, TasP Home services manager 1)*

Besides, several TasP HCWs reported circumstances in which their services were not appreciated. Some people refused services, claiming that they needed more help than the trial protocol provided (e.g. food or transport to go to the clinic). Others lied about their identity to avoid being offered services. And others expressed their annoyance or dislike over repeated home services visit, specifically regarding repeat HIV testing, every six months.

*“Sometimes, when you get inside [a participant’s house], [a family member] will come after you have introduced yourself. And s/he will ask: “Who do you want to speak with?” Then s/he will say*: *“S/he is not here.” Then you will find out that this person you are talking to is actually the one you are looking for (to offer him/her testing). And then the whole family will laugh at you and you will end up looking like a fool.” (SSI, TasP home linkage-fieldworker 1)*

*“It happened that you visit the house of someone that has already tested positive for HIV in a previous trial round. So s/he will say: “You have found me last time and tested me positive*. *So, what are you here to do now? I now know my status. There is no need to keep coming back to me. You should continue with others who have not tested yet or those who are not positive.” (FGD, TasP home testing-fieldworkers, participant 9)*

### Home-based services do not always preserve full confidentiality and freedom of choice

Some TasP HCWs reported during interviews having witnessed people’s discomfort and refusal of HIV home services. Certain people feared being seen in contact with an institution working on HIV; their neighbours would label them as living with HIV. In the quantitative study, over half of HCWs expressed concerns about a possible lack of confidentiality of home-based HIV services (Fig 2). Such a result was explained by HCWs as the fact that, during home-based HIV testing, unintentional HIV status disclosure could occur in the absence of soundproof rooms in poor households, or because time of counselling may be longer for people with a positive HIV diagnosis, or also because a positive HIV diagnosis could be read on people’s faces. Regarding support for linkage and retention in HIV care, a confidentiality breach could happen as the services specifically target people living with HIV.

*“If people are tested at the clinic, they receive their results there and cry all the way home. And they arrive at home in a good state. But if you test them at home, some just fail to act as if they are okay*. *When s/he goes out of the room, you can just see that s/he is not okay. And it means that things did not go well inside. So, at home the confidentiality might be compromised.” (FGD, TasP home testing-fieldworkers, participant 3)*

Some TasP HCWs who delivered services at home mentioned the important role that family members could play in people’s acceptance of health services. Individuals could feel forced into accepting or refusing services. TasP HCWs talked about the discomfort of standing out of the family crowd, and the fear of feeling obligated to report to the family on the outcome of the discussion with the HCW, especially among young adults and women who live under their husband’s control.

*“The other challenge that [young people] have about testing at home, is that if they test*, *parents or old people want to know his/her results when s/he is done.” (FGD, TasP home testing-fieldworkers, participant 5)*

Further, some TasP HCWs raised the issue of possible coercion of people by HCWs into accepting home-based HIV services.

*“Some community members feel forced by the home linkage-fieldworkers to come [to the clinic]. There is a slim chance that those people will then continue attending care*. *Indeed, you put them in class at the first clinic visit where they learn. But then they leave and never come back to the clinic, because they were forced to come at first. They just came so that anyone who was on their case (referring to the home linkage-fieldworkers) would just leave them alone.” (FGD, TasP clinic ART counsellors, participant 1)*

Nevertheless, the quantitative study showed that more than four out of five HCWs (87%) disagreed with the fact that home-based HIV testing could force people into accepting services (Fig 2).

#### Home-based services entail challenging working conditions

Several TasP HCWs mentioned how they were emotionally affected while assisting very poor and disadvantaged people with HIV testing or support for linkage and retention to care services.

*“A thing that I can say was a bit challenging was when I found a participant who was sick and I failed to assist him/her. […] S/he was sick and s/he had no means to get to the clinic*. *[…] I had to leave that participant sleeping there at his/her home. S/he was not able to walk to the clinic and I was not allowed to bring him/her there in our trial cars.” (SSI, TasP home-linkage-fieldworker 2)*

TasP HCWs also narrated unpleasant, and even scary, situations they faced while delivering services in the community. All TasP HCWs talked about the daily operational challenges, which result from working in a rural, poor and remote area with scattered households. Many of them regretted that vehicles were sometimes unreliable, despite them being essential to reach people’s homes. Moreover, some explained that they feared for their safety (e.g. dog bites, physical aggressions). They often held the line management accountable for their low training and operational support.

*“Some community members in the community that I was working with, told me […] that they wanted to rob me and they wanted to even take the material that I worked with*. *They wanted to abuse me because I kept on coming to do the one and the same thing. I was in danger and had to make sure that when I walked, I walked with males and not in dark or isolated places.” (SSI, TasP home linkage-fieldworker 7)*

Additionally, government HCWs regretted the lack of an established policy framework for home-based services.

*“There are [government] clinics that have an outreach team which is a team with a sister* (meaning a professional nurse), *CCGs and some nurses that visit people at home*. *But in our clinic*, *we don’t have such a team*. *We are hoping that*, *in the future*, *we are going to have one because*, *in the establishment* (meaning in the official health district programme), *it does state that there should be an outreach team*, *but we still don’t have it*. *[…] I think the Hlabisa department of health has a problem with transport*. *It seems that they don’t have enough cars*. *But each outreach team needs a transport*.*” (SSI*, *Government clinic nurse 2)*

### Home-based HIV services promote re-thinking the current model of care towards differentiated care

#### Support for the integration of home-based HIV services in the local health system, despite organizational challenges

All HCWs supported the further implementation of home-based services for HIV testing and support for linkage and retention in care as part of the local model of care. Some government HCWs also shared their willingness to participate in the home activities if given the opportunity.

*“If you are here at the clinic, you do not know how many people need help in the community. I think we do need to increase mobile clinics that visit people at home*. *[…] Because we will also like to work within homes, but we can’t because we must be here at the clinic” (SSI, government clinic nurse 1)*

In the quantitative study, a large majority of HCWs expressed their support for the implementation of yearly HIV testing campaigns (95%) and a linkage to care services programme (86%) (Fig 2).

Some HCWs were also convinced that the integration of home-based HIV testing in the local health system would participate in decreasing HIV-related discrimination and normalizing regular HIV testing, as all households would be visited.

*“I think the HIV-related discrimination could be decreased with home-based HIV testing because participants could see the importance* of testing *[…] Even if they don’t like being tested for HIV*, *they will do it*, *simply because it is done to the whole community*. *They will not want to be the only one who is not doing it*.*” (FGD*, *TasP home testing-fieldworker*, *participant 7)*

Yet, many HCWs pointed out organizational challenges that would ensue from the integration of home-based HIV services in the local HIV programme. In the quantitative study, most participants expressed concern about a lack of human resources for implementing home-based services in the local health system (93% regarding HIV testing and 86% regarding support for linkage (Fig 2)). During the interview sessions, HCWs formulated advice for implementers: involve local leaders (“izindunas” and “ward councillors”) to inform the community on the programme, dedicate enough financial means, hire a big enough workforce and provide them with proper means (e.g. transport, equipment), adequate training and continuous on-the-job support and officially recognize the value and burden of home-based work.

#### Beyond business as usual: integrated HIV care outside of the clinic walls

All TasP home testing-fieldworkers reported during the interviews that they were regularly asked by trial participants for additional services at home such as other HIV-related services (e.g. delivery of ART), sexual health services (e.g. pregnancy diagnosis, screening of sexually transmitted infections) and/or chronic care services, suggesting that home-based care could be an opportunity for the offer of a whole range of integrated services, beyond HIV, especially for poor and disadvantaged people. This result was corroborated by the quantitative study with a strong majority of participants supporting the introduction of a multi-diseases screening (86%) and ART initiation (85%) during home visits (Fig 2).

*“Many people who agree to be part of the [TasP] study are old people, grandmothers, grandfathers and aunts […] So if the government starts this programme of testing participants at home, it should also introduce blood pressure and diabetes [screening] so that it can also help old people*.*” (FGD, TasP home testing-fieldworkers, participant 3)*

Moreover, many HCWs were in favour of pairing home-based services with community and clinic-based services. They repeatedly talked about the specificity of certain population groups who are responsive to certain delivery methods, at certain moments, in certain contexts; suggesting that a panel of HIV services would allow for a better response to the diversity of healthcare needs. HCWs explained how community-based services would be particularly adequate in this rural area, for HIV testing and support for linkage and retention in care: provided in tents or mobile clinics, strategically located in crowded community places like schools or community halls, possibly during social events such as sport tournaments, stage plays or on the days of national grants retrieval. Within the quantitative study, 71% of the participants agreed that HIV testing in mobile units would be acceptable by the population they serve (Fig 2).

*“What can assist in attracting the youth to test is to test them in mobile units, in sports-grounds and in places where there are events. If you find them in a sports-ground or anywhere where they can come with their friends*, *[then they can] test without everyone from home.” (FGD, home-testing fieldworkers, participant 5)*

*“The school children do not want to come [in the clinics for HIV testing]*. *If maybe there could be teams who visit schools [to offer HIV testing], just as X (referring to a government clinic situated in the trial area) did. X contributed a lot because it tested children at school.” (SSI, government clinic nurse 3)*

Overall, HCWs were in favour of moving HIV care outside the clinic walls while pairing it up with other health services, tailored to address the national burden of disease.

## Discussion

This study explored, in the context of a large-scale community-based trial conducted in rural South Africa, the views of HCWs towards HIV services delivered at people’s home: HIV testing, support for linkage to HIV care, and support for retention in HIV care. Our findings overall highlight that, regardless of whether they themselves delivered HIV services to people’s homes, or whether they reported positive or negative field experiences, all HCWs considered the delivery of services at home as feasible and acceptable by the population they serve. Similar findings were noted in quantitative and qualitative studies examining home-based HIV services delivery in sub-Saharan Africa [40–43]. HCWs thus supported home-based services as one of the key components of a comprehensive UTT strategy to benefit people’s health.

Our results demonstrated that home-based HIV testing plays a key role in ensuring high HIV testing coverage within rural communities, especially in underserved areas. This positive qualitative assessment of the HIV testing services is supported by the trial quantitative results, as the vast majority of the trial population (92%) accepted to be tested for HIV at home [31]. Such analysis is confirmed by other quantitative [40, 43–45] and qualitative studies [46] that examined the impact of home-based testing on HIV testing coverage in sub-Saharan Africa. HCWs also underlined that testing people at home provide opportunities to identify people living with HIV early in their infection, supporting its potential to assist a UTT strategy: the earlier a person gets to know about his/her status, the sooner he/she can be introduced to ART, and subsequently improve his/her own health and prevent the transmission of the HIV infection. Besides, a growing literature on cost-effectiveness of home-based services including HIV testing, outline the relevance of moving outside of the clinic walls to improve people’s health [47]. These findings are in line with the UNAIDS targets, outlining the importance of increasing the number of people who are aware of their HIV status.

HCWs highlighted the positive influence home visits have on increasing access to HIV care in the community, regardless of the type of services delivered. HCWs have shown potential to assist people in navigating the HIV care trajectory from their home to the clinic [46, 48]. Our analysis thus confirms the capacity of home-based services to encourage people to seek healthcare, while reducing the burden on the facility-based health system. Those results are supported by previous quantitative and qualitative analysis in the TasP trial that show that home visits for HIV testing and support for linkage and retention in care participate in encouraging people to initiate ART early [49, 50]. Such findings are complemented by those of other studies in sub-Saharan Africa that make apparent the key role of home-based services in improving and sustaining ART initiation and adherence [44, 51–53].

However, some of the disadvantages of home-based services were revealed by our findings. Some population groups are left behind by home-based services, including young adults and men [11, 43, 54]. Further research is required to adapt home services to such vulnerable populations. Respondents also outlined that home-based services should rather be implemented as complements to the existing facility and community-based services, rather than as standalone strategies. Such findings build on the plea for differentiated and person-centred care to reach the UNAIDS targets, especially in resource-constrained settings with high burdens of HIV infection [55].

Our study reports on several of the specificities of providing health services outside of health centres, in people’s homes. First, respondents described the underlying fear of status disclosure and stigma happening within households. As initiation and maintenance of people’s HIV care trajectories have been shown to be influenced by family support [56–58], our study confirms the key role of a caring steady family environment in the success of one’s home-based care. HCWs therefore stand in favour of safeguarding confidentiality and preventing possible coercion, discrimination and other adverse consequences for populations being offered services in their homes. Second, HCWs reported on the specificities of provider-patient interactions at people’s homes, mainly related to the nature of home-based HIV services. Such findings add up to those of other studies, noting that HCWs engaged in extended one-to-one talks outside of the comfort-zone of a clinic, and formed professional and social relationships with their patients [59–63]. Such a privileged relationship could inspire action in people and provide them with support to take up and maintain access to HIV services.

HCWs reported on the demands emerging from community members, and themselves argued, that home-based approaches for HIV care could be leveraged as a conduit for delivering care beyond testing and support. Such findings support ART initiation, delivery and monitoring at people’s homes, which have been shown to be feasible and acceptable in sub-Saharan African countries [52, 53, 64] and are currently being considered as promising innovations in South Africa [28] and other high-prevalence settings [65]. In response to the national burden of disease, the use of home-based approaches to address other long-term conditions beyond HIV has also been widely encouraged by community members. Such a stance is supported by the recent multi-diseases home-based interventions which have been investigated in sub-Saharan Africa, with great success in terms of acceptability [66, 67] and indications of likely cost-effectiveness [18, 68], and which are also in the pipeline of national health strategies, including in South Africa [28]. With the international focus on Universal Health Coverage [69, 70], home-based interventions seem attractive to reshape the disease specific and care-oriented services towards more comprehensive goals [71, 72]. But the features of such an integrated health system approach remain to be defined, including the careful selection of the services to benefit the health of family members and not overburden them unnecessarily. Substantial organizational efforts are expected for the implementation of standardized and yet context-specific solutions [73, 74].

Government HCWs, in addition to adopting a positive position for the scale up of home-based HIV services in the Hlabisa area, insist on the lessons learnt from the current local home-based care programme. They value the support provided by CCGs to the government clinics but note the poor supervision and irregular geographical distribution. Such findings support the formalizing of home-based care (Box 1) in line with the current efforts undertaken in several sub-Saharan African countries including in South Africa [28]. Government and TasP staff unite to plea for adequate training and support of HCWs (Box 1), adding to the growing claim of the health profession in sub-Saharan African countries for training opportunities that fully embrace the broad spectrum of competencies and cadres, in order to effectively meet health needs [75, 76]. Government and TasP staff also pointed out how an official recognition of the specific value and burden of their work could participate in improving the delivery of services in people’s homes (Box 1), in keeping with the words of other HCWs who delivered home-based services, including HIV services in South Africa [77]. Those findings come at a time when the last decade has highlighted major gaps in the availability, accessibility and quality of the health workforce in many countries [78–80] and home-based HCWs represent an opportunity to shape the development of a new workforce and rationalize health care demands [81, 82]. Based on the insights of HCWs, we formulated pragmatic recommendations to policy-makers, to implement and improve the delivery of home-based services as a key component of national health systems (Box 1).

**Box 1. Recommendations to implement and improve the delivery of HIV services at home, in rural areas: lessons learnt from HCWs**

○ Policy implementation: To formalize the delivery of home-based services
  ◼ To mandate home-based services as part of HIV care, within all pertinent program guidelines
  ◼ To develop practice standards at national, district and sub-district levels that are adapted to people’s needs (home visits outside of working hours, phone calls prior home visits, mechanisms to transport people from home to clinics)
  ◼ To drive policy-makers into understanding the distinctiveness of home-based work and challenge them to make long-term commitment to funding home-based programs
○ Health services management: To build a home-based workforce
  ◼ To recruit a specific workforce to be assigned to home visits
  ◼ To integrate and articulate the workforce into the existing clinic-based pool staff
  ◼ To provide the workforce with strong management and supervision, including allowing for feedback mechanisms
○ Human resources: To adequately train and build the skills of the home-based workforce
  ◼ To manage psychological aspects encountered during home visits (confidentiality, coercion, discrimination, especially with vulnerable population groups (e.g. men, young adults))
  ◼ To build a cultural sensitivity to really get the value of home-based work through the building of privileged provider-patient interactions

One of the limitations of the quantitative part of the study was that questionnaires were written in English. This may have prevented the staff members who were less proficient in English from fully understanding the questions. Piloting the questionnaires prior the onset of data collection helped change the wording of some questions to minimize misunderstandings. One of the limitations of the qualitative part of the study is that participants may have responded in a way that they felt was expected as researchers who carried out the analysis were also working within the TasP trial. For some health care workers delivering home-based services in the TasP trial, this study allowed them to reflect on their own activity, which may have biased their response in one way or another. We noticed signs of irritability when they felt that the quality of their work was questioned and we assumed that they might have been reluctant to make critical responses. Additional training was given to the qualitative interviewers on probing to capture a full range of experiences. We ensured anonymity and created a space for open critical discussion, by open-ended questioning, and critical responses did emerge. Additionally, we aimed at an in-depth understanding of the context-specific issues through a close collaboration between isiZulu-speaking qualitative interviewers and researchers with an extensive knowledge of the UTT strategy of the TasP trial. Finally, even though our study results may not be generalizable outside of the context of clinical trials and outside of South Africa, they bring valuable insights to understand the challenges of HIV service delivery in rural KwaZulu Natal, one of the most HIV-affected areas in the world; our results also provide informative lessons for contexts that are similar to our study setting (weak health systems serving particularly vulnerable populations living in dispersed settlements and greatly affected by HIV).

This study is, to our knowledge, the first investigating home-based services in the framework of a UTT strategy. The mixed-methods design allowed a comprehensive investigation process of the situation. By and large, many of the difficulties of formalizing and strengthening home-based care are currently being investigated. This paper seeks to place these challenges in the South African context, which is rapidly evolving with respect to changing health needs: all people living with HIV are now eligible for HIV treatment. Our analysis confirms that home-based HIV services hold a prominent place in the UTT arsenal to improve the overall care cascade. The central challenge appears to lie less in technical systems design than in making a case for a home-based system to be a part of the established health system.

## Supporting information

S1 Table Topics mentioned by health care workers during the qualitative study (ANRS 12249 TasP trial, Hlabisa sub-disctrict, South Africa, 2014). IDI: in-depth interview, FGD: focus group discussion.
(DOCX)
