# Supplementary material for: “If you are here at the clinic, you do not know how many people need help in the community”: Perspectives of home-based HIV services from health care workers in rural KwaZulu-Natal, South Africa in the era of universal test-and-treat
Source: PLoS One. 2018 Nov 9;13(11):e0202473. doi: 10.1371/journal.pone.0202473 (PMC6226311; doi:10.1371/journal.pone.0202473)
Supplement: S1 Table — IDI: in-depth interview, FGD: focus group discussion. (DOCX) [file pone.0202473.s001.docx]

Supporting information

**S1 Table. Topics mentioned by health care workers during the qualitative study (ANRS 12249 TasP trial, Hlabisa sub-disctrict, South Africa, 2014)**

|  | IDI - TasP Home linkage-fieldworker 1 | IDI - TasP Home linkage-fieldworker 2 | IDI - TasP Home retention-fieldworker | IDI - TasP Home services manager 1 | IDI - TasP Home services manager 2 | IDI - TasP Clinic nurse 1 | IDI - TasP Clinic nurse 2 | IDI - TasP Clinic nurse 3 | IDI - TasP Clinic nurse 4 | IDI - TasP Clinic ART counsellor 1 | IDI - TasP Clinic ART counsellor 2 | IDI - TasP Clinic trial manager 1 | IDI - TasP Clinic trial manager 2 | IDI - Government Clinic nurse 1 | IDI - Government Clinic nurse 2 | IDI - Government Clinic nurse 3 | IDI - Government Clinic services manager 1 | IDI - Government Clinic services manager 2 | FGD – TasP Home testing-fieldworkers | FGD – TasP Clinic nurses | FGD – TasP Clinic ART counsellors |
| --- | --- | --- | --- | --- | --- | --- | --- | --- | --- | --- | --- | --- | --- | --- | --- | --- | --- | --- | --- | --- | --- |
| 1. **Home-based services have the potential to draw into care people who have slipped through the cracks of the existing health care system** | | | | | | | | | | | | | | | | | | | | | |
| - 1. Home-based services are perceived as highly acceptable and very convenient. | | | | | | | | | | | | | | | | | | | | | |
| - - 1. People appreciated receiving HIV services at home. | x | x |  |  |  |  |  |  |  |  | x |  |  |  |  |  |  |  | x |  |  |
| - - 1. Home-based services allowed to overcome common structural and individual barriers to facility-based health services. |  | x |  | x | x |  |  |  |  |  |  |  |  |  | x |  |  |  | x |  |  |
| - 1. Home-based services enable a strong support for HIV care due to family closeness and connectedness with HCWs. | | | | | | | | | | | | | | | | | | | | | |
| - - 1. The family is central in people’s acceptance of HIV services at home. | x |  | x |  |  |  |  |  |  |  |  |  |  |  |  |  |  |  | x |  |  |
| - - 1. HCWs display strong interpersonal skills to perform services at people’s homes. | x | x |  | x | x |  |  |  |  |  |  |  |  |  |  |  |  |  | x |  |  |
| - 1. Home-based services are conducive to promoting entry and retention in HIV care. | | | | | | | | | | | | | | | | | | | | | |
| - - 1. Home-based HIV testing allow people to learn about their positive HIV status early |  |  |  |  |  |  | x | x | x |  |  |  |  |  | x | x |  |  | x |  | x |
| - - 1. Personalized support at home encourage people to enter and be retained in care. |  | x |  | x |  |  |  | x |  |  |  |  |  |  |  | x |  | x | x |  | x |
| - - 1. Respectful HIV services at home contributed to building people’s trust towards clinic-based health care. |  |  |  |  |  |  |  |  |  |  |  |  |  |  |  | x |  |  |  |  |  |

IDI: in-depth interview, FGD: focus group discussion

S1 Table (ctd.). Topics mentioned by health care workers during the qualitative study (ANRS 12249 TasP trial, Hlabisa sub-district, South Africa, 2014)

|  | IDI - TasP Home linkage-fieldworker 1 | IDI - TasP Home linkage-fieldworker 2 | IDI - TasP Home retention-fieldworker | IDI - TasP Home services manager 1 | IDI - TasP Home services manager 2 | IDI - TasP Clinic nurse 1 | IDI - TasP Clinic nurse 2 | IDI - TasP Clinic nurse 3 | IDI - TasP Clinic nurse 4 | IDI - TasP Clinic ART counsellor 1 | IDI - TasP Clinic ART counsellor 2 | IDI - TasP Clinic trial manager 1 | IDI - TasP Clinic trial manager 2 | | IDI - Government Clinic nurse 1 | IDI - Government Clinic nurse 2 | IDI - Government Clinic nurse 3 | IDI - Government Clinic services manager 1 | IDI - Government Clinic services manager 2 | FGD – TasP Home testing-fieldworkers | FGD – TasP Clinic nurses | FGD – TasP Clinic ART counsellors |
| --- | --- | --- | --- | --- | --- | --- | --- | --- | --- | --- | --- | --- | --- | --- | --- | --- | --- | --- | --- | --- | --- | --- |
| 1. **Home-based services are not a magic bullet** | | | | | | | | | | | | | | | | | | | | | | |
| - 1. Home-based services don’t reach everyone |  |  |  |  |  |  |  |  |  |  |  |  |  |  |  |  |  |  |  |  |  |  |
| - - 1. People were not present in their households at the time of the home visits. |  |  |  |  |  |  |  |  |  |  |  |  |  | |  |  |  |  |  | x |  |  |
| - - 1. Some people refused services. |  |  |  |  |  |  |  |  |  |  |  |  |  | |  |  |  |  |  | x |  |  |
| - 1. Home-based services do not always preserve full confidentiality and freedom of choice. | | | | | | | | | | | | | | | | | | | | | | |
| - - 1. People feared being seen in contact with an institution working on HIV. | x | x |  |  | x |  |  | x |  |  |  |  |  | |  |  |  |  |  | x |  |  |
| - - 1. Unintentional HIV status disclosure could occur. | x |  |  |  |  |  |  |  |  |  |  |  |  | |  |  |  |  |  | x |  |  |
| - - 1. Possible coercion of people by their family members into accepting or refusing home-based HIV services. | x | x | x |  | x |  |  |  |  |  |  |  |  | |  |  |  |  |  | x |  |  |
| - - 1. Possible coercion of people by HCWs into accepting home-based HIV services. |  |  |  |  |  |  |  |  |  |  |  |  |  | |  |  |  |  |  |  |  | x |
| - 1. Home-based services entail challenging working conditions. | | | | | | | | | | | | | | | | | | | | | | |
| - - 1. HCWs are emotionally affected while assisting very poor and disadvantaged people. |  | x |  |  |  |  |  |  |  |  |  |  |  | |  |  |  |  |  | x |  |  |
| - - 1. HCWs experienced unpleasant, and even scary, situations while delivering services in the community | x | x |  |  |  |  |  |  |  |  |  |  |  | |  |  |  |  |  | x |  |  |
| - - 1. There is a lack of an established policy framework for home-based services in the current government services |  |  |  |  |  |  |  |  |  |  |  |  |  | |  |  |  |  | x |  |  |  |

IDI: in-depth interview, FGD: focus group discussion

S1 Table (ctd.). Topics mentioned by health care workers during the qualitative study (ANRS 12249 TasP trial, Hlabisa sub-district, South Africa, 2014)

|  | IDI - TasP Home linkage-fieldworker 1 | IDI - TasP Home linkage-fieldworker 2 | IDI - TasP Home retention-fieldworker | IDI - TasP Home services manager 1 | IDI - TasP Home services manager 2 | IDI - TasP Clinic nurse 1 | IDI - TasP Clinic nurse 2 | IDI - TasP Clinic nurse 3 | IDI - TasP Clinic nurse 4 | IDI - TasP Clinic ART counsellor 1 | IDI - TasP Clinic ART counsellor 2 | IDI - TasP Clinic trial manager 1 | IDI - TasP Clinic trial manager 2 | IDI - Government Clinic nurse 1 | IDI - Government Clinic nurse 2 | IDI - Government Clinic nurse 3 | IDI - Government Clinic services manager 1 | IDI - Government Clinic services manager 2 | FGD – TasP Home testing-fieldworkers | FGD – TasP Clinic nurses | FGD – TasP Clinic ART counsellors |
| --- | --- | --- | --- | --- | --- | --- | --- | --- | --- | --- | --- | --- | --- | --- | --- | --- | --- | --- | --- | --- | --- |
| 1. **Home-based HIV services promote re-thinking the current model of care towards differentiated care** | | | | | | | | | | | | | | | | | | | | | |
| - 1. Support for the integration of home-based HIV services in the local health system, despite organizational challenges | | | | | | | | | | | | | | | | | | | | | |
| - - 1. Reasons to support the implementation of home-based services in the local model of care. | x | x | x | x | x |  |  |  |  |  |  |  |  |  |  | x |  | x | x |  |  |
| - - 1. Organizational challenges that would ensue from the integration of home-based HIV services in the local HIV programme. | x |  | x | x | x |  |  |  |  |  |  |  |  |  |  |  |  |  | x |  | x |
| - - 1. Advice for implementers to implement home-based services at a large scale. | x | x | x |  |  | x | x | x |  | x |  | x | x | x | x | x | x |  |  | x | x |
| - 1. Beyond business as usual: integrated HIV care outside of the clinic walls | | | | | | | | | | | | | | | | | | | | | |
| - - 1. Possible additional services to be delivered at home | x | x | x | x |  |  |  |  |  |  |  |  |  |  |  |  | x |  | x |  |  |
| - - 1. Possible to pair home-based services with community and clinic-based services. |  |  |  | x | x |  |  |  |  |  |  |  |  |  |  |  |  |  | x |  |  |

IDI: in-depth interview, FGD: focus group discussion
